# Capillary flow-driven microfluidic device with wettability gradient and sedimentation effects for blood plasma separation

**DOI:** 10.1038/srep43457

**Published:** 2017-03-03

**Authors:** M. Sneha Maria, P. E. Rakesh, T. S. Chandra, A. K. Sen

**Affiliations:** 1 Department of Mechanical Engineering, Indian Institute of Technology Madras, Chennai-600036, India; 2 Department of Biotechnology, Indian Institute of Technology Madras, Chennai-600036, India

## Abstract

We report a capillary flow-driven microfluidic device for blood-plasma separation that comprises a cylindrical well between a pair of bottom and top channels. Exposure of the well to oxygen-plasma creates wettability gradient on its inner surface with its ends hydrophilic and middle portion hydrophobic. Due to capillary action, sample blood self-infuses into bottom channel and rises up the well. Separation of plasma occurs at the hydrophobic patch due to formation of a ‘self-built-in filter’ and sedimentation. Capillary velocity is predicted using a model and validated using experimental data. Sedimentation of RBCs is explained using modified Steinour’s model and correlation between settling velocity and liquid concentration is found. Variation of contact angle on inner surface of the well is characterized and effects of well diameter and height and dilution ratio on plasma separation rate are investigated. With a well of 1.0 mm diameter and 4.0 mm height, 2.0 μl of plasma was obtained (from <10 μl whole blood) in 15 min with a purification efficiency of 99.9%. Detection of glucose was demonstrated with the plasma obtained. Wetting property of channels was maintained by storing in DI water under vacuum and performance of the device was found to be unaffected over three weeks.

Blood is the most important biological fluid which stands as the medium for transporting nutrients, oxygen and immune cells throughout the body and also maintains body temperature and pH. It is also an active indicator of various pathological disorders^1^. Analysis of the cells in blood, the occurrence of viral or bacterial components and the imbalances in the composition of blood can be used for the detection of diseases and organ malfunctioning^2^. Blood is a suspension of various formed elements (45% by volume of whole human blood) in plasma. The presence of these formed elements such as red blood cells (95% of total formed elements), white blood cells (0.13%) and platelets (4.5%) impart a non-Newtonian property to the blood. Red blood cells (RBCs) have a biconcave shape with diameter 6 to 8 μm and thickness 1.5 to 2 μm. Plasma is a straw coloured liquid which consists of various proteins such as fibrinogen, globulin and albumin in water (95%) and is slightly viscous than water^3^. Efficient separation of plasma (plasmapheresis) is the first step for the analysis of blood in most of the diagnostic studies^4^.

The conventional process of plasma separation, i.e. centrifugation, is performed in macroscale which requires several millilitres of blood and bulky apparatus. For patients who need regular monitoring of blood, such a procedure is highly inconvenient due to the high volume of sample blood and transportation time required (to the place where the setup is installed). For example, in the case of blood glucose monitoring (BGM) in acute diabetic patients, the glucose level may vary with time during a single day. So a portable, self-operable device which uses microscale quantity of blood is the need of the hour which could make the primary diagnostic tests simple, accessible and cost effective. In microfluidics, active^5^,^6^,^7^ as well as passive^8^,^9^,^10^,^11^,^12^,^13^,^14^,^15^ methods have been developed for the separation of plasma from sample blood. In addition to this, paper^16^,^17^ and Compact Disc (CD)-based^18^,^19^ methods for blood plasma separation are also reported. In active methods, acoustic, electrical or magnetic fields are used to impart sufficient energy for the blood plasma separation, which makes the entire system bulky and complicated^20^. Although active devices are efficient in terms of purification efficiency, such devices offer lower residence time and throughput^21^. The passive devices operate based on the principles of hydrodynamic forces and response of cells to various biophysical effects. Such devices are advantageous due to simpler design, ease of fabrication, continuous operation and low cost^22^. The various techniques used in the passive devices include sedimentation, microfiltration and hydrodynamic forces developed due to the mechanical properties of particles^23^. A major challenge in the blood plasma separation devices in general is to limit the stress acting on the cells, which causes haemolysis *in-vitro* thus hindering diagnosis. In this context, the passive devices perform better than active devices and offer higher cell viability^24^. However, both active as well as passive devices require pumps for the infusion of sample blood into the device which limits its use for Point-of-Care (POC) applications.

Use of capillary force as the driving force and sedimentation and filtration as separation mechanisms enable simpler design and thus suitable for point of care (POC) applications. A surfactant-added PDMS device based on capillary flow phenomenon and cross-flow filtration was reported for the extraction of plasma from sample without any external driving force^25^. A microfluidic device which employed asymmetric capillary flow, which was realized by surface modification of one of the channel walls using spray coating of silica nanobead multilayers, was used for blood plasma separation^26^. In both these cases, the plasma recovery of the device is only 3.4% and the use of surfactant or silica nano-assembly makes the device fabrication very challenging and expensive. Degassing of channel in the microfluidic device using vacuum desiccator to enable the flow of sample blood into the microchannel device was reported^27^,^28^. In ref. 27, a two layered PDMS chip in which a membrane filter is placed on the top of the vertical channel was used to separate plasma as blood rises up the channel with the additional effect of gravitation force acting on the settling cells. In ref. 28, plasma separation is demonstrated in a cylindrical PDMS channel where the sample blood while flowing from the bottom of the channel sediment and the separated plasma flows upward. The work reports mainly pump-driven plasma separation and one set of experiment with vacuum assisted plasma separation is also presented. However, this study uses diluted blood as sample and the volume of plasma separated is not reported. Although, these techniques offered higher plasma recovery, the sample blood had to be introduced into the device immediately after the removal of the device from the vacuum. The time gap between the time instants when the device is taken out of the vacuum chamber and when the sample blood is introduced into the device affects the performance of the device, which limits its use in POC applications. Due to the growing need of capillary flow in healthcare and diagnostic applications, attempts have also been made to model the capillary flow of non-Newtonian fluids. Cito *et al*.^29^ generalized the Lucas-Washburn equation for the flow of power-law non-Newtonian fluids through a straight channel and Digilov^30^ has developed a relation to predict the evolution of meniscus height in a vertical channel with time for non-Newtonian liquids. Siddique and Anderson^31^ have explored the capillary rise of a non-Newtonian, power-law fluid into rigid and deformable porous materials with and without gravity effects.

Here, we present a two layered (top and bottom) microchannel device with a vertical cylindrical well connecting the bottom and top straight rectangular channels. Exposure of the substrate containing the vertical cylindrical well to oxygen plasma creates varying wettability on the inner surface of the well with the ends of the well more hydrophilic and middle portion acting as a hydrophobic patch. Separation of plasma from whole blood takes place at this hydrophobic patch region due to the formation of a self-built-in filter and by sedimentation. The device is simpler, easier to fabricate, less expensive and provides higher plasma recovery as compared to capillary based blood plasma separation devices reported in literature. First, a brief description of the device and the operating principle is outlined. Further, fabrication of the device, experimental setup and procedure are outlined. Finally, results of the experiments and model in terms of blood flow, RBC sedimentation and blood plasma separation are presented and discussed.

## Device Description and Principle

**Figure 1:**
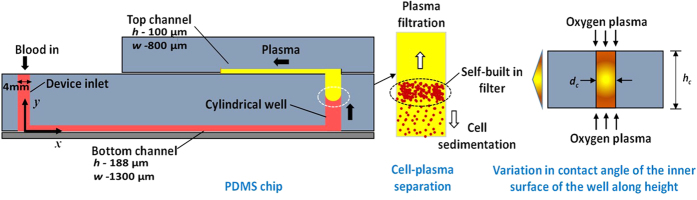
Schematic of the device structure, plasma filtration due to moving self-built in filter and sedimentation shown, variation of contact angle inside the cylindrical channel (well) due to oxygen plasma exposure is also shown.

A schematic of the proposed blood plasma separation device is shown in Fig. 1. The device has two rectangular microchannels: top and bottom microchannels, which are connected by a vertical cylindrical channel, the well. A cylindrical port serves as the inlet to the device for introducing the sample blood. Except the vertical cylindrical channel (i.e. the well), all other channels are uniformly hydrophilic on all sides. When the PDMS substrate containing the well is exposed to oxygen plasma, both ends of the well get well exposed to plasma. However, plasma exposure at the middle portion of the well is limited due to a reduced penetration of oxygen plasma into the well. Further, it leads to varying hydrophilicity along the height of the well with both ends of the well more hydrophilic and the central portion less hydrophilic (or hydrophobic), which behaves as a hydrophobic patch, as depicted in Fig. 1.

When sample blood is introduced at the device inlet, due to a difference in the Young- Laplace pressure, it flows through the bottom hydrophilic rectangular channel by capillary action and arrives at the bottom of the well. As the blood rises up the well, on account of the increasing contact angle at the hydrophobic patch near the central portion, the capillary force decreases thus significantly slows down the flow. First of all, as the capillary flow velocity decreases with rise in meniscus height, the cells tend to sediment due to their higher density while the blood plasma rise up the well by capillarity due to its significantly lower viscosity (about 2.5 times^32^). By this combined effect of gravity and capillarity, separation of plasma from blood commences. At the hydrophobic patch region, when the blood flow is impeded, RBCs tend to accumulate and sediment thus act as a moving self-built in filter (screen) to facilitate the separation of plasma. Since the viscosity of plasma is significantly lower than that of the blood cells, the plasma moves at a higher velocity as compared to the blood cells in the hydrophilic region. Thus, the blood plasma flows (up) past the moving (down) self-built in filter and gets separated from the blood cells. Finally, the separated plasma flows into the upper microchannel, where detection of analytes could be performed.

## Device Fabrication and Experimental Setup

The straight microchannels at the top and bottom of the device were fabricated as described in the Supplementary Information whereas the vertical channel (well) was created by punching holes in the bottom PDMS substrate using biopsy punches (Shoney scientific, Pondicherry, India) of sizes 0.75 mm, 1.0 mm, 1.5 mm and 2 mm. The top PDMS layer with a 800 × 100 μm channel and the bottom PDMS layer with a 1300 × 188 μm channel and the vertical cylindrical channel (well) were placed inside a plasma chamber (Harrick Plasma, Brindley St., USA) separately with the channel of the top layer facing up and channel in the bottom layer facing down. The layers were then exposed to oxygen plasma of power 11.0 W for 2 min at a chamber pressure of 420 m.Torr and bonded with each other such that the well opening in the bottom layer and the top channel are aligned. Next, a second plasma exposure step was performed with the bottom channel of the bonded assembly facing up and a glass slide which were then bonded by applying gentle pressure. So the vertical channel (well) gets exposed to oxygen plasma equally from its both ends and the hydrophilicity is symmetrical about the centre portion. Since the centre portion of the well gets less exposed to the oxygen plasma, it is less hydrophilic as compared to the both ends and thus a wettability gradient is achieved. Since the diameter of the device inlet port is much higher (>3 mm) as compared to that of the vertical channel (well), the centre portion of the inlet port gets well exposed to plasma. Also, since the blood sample is placed into the inlet port using pipette and it falls to the bottom of the port due to gravity, presence of any minor variation in the wettability in the inlet port does not have an effect. In order to clearly observe the plasma separation inside the vertical cylindrical channel, the rough sides of the devices were coated with PDMS and cured to improve optical quality. In order to observe static sedimentation, cylindrical wells were prepared in PDMS blocks using 1.5 mm punch.

**Figure 2:**
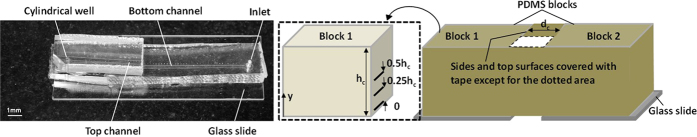
Photograph of the (**a**) proposed device (**b**) schematic showing the arrangement of PDMS blocks and (right) positions of the blood drops on the inner face of the block (right) for the contact angle studies.

To simulate the variation of contact angle on the inner surface of the well along the height, PDMS blocks of width 3.0 mm and different heights, 4.0 mm, 6.0 mm and 8.0 mm were prepared. Two blocks of same height were placed adjacent to each other with different gaps i.e. 0.75 mm, 1.0 mm and 2.0 mm in between, which corresponds to the diameters of the vertical channels (Fig. 2b). Only the top and bottom sides was open to allow oxygen plasma (through a square opening) and all other sides of PDMS blocks were sealed with a cello tape to ensure that the oxygen plasma does not enter from the sides but only through the top and bottom sides similar to that in the actual device. The setup (Fig. 2b) was exposed to oxygen plasma from both top and bottom sides and disassembled for contact angle measurements. Five different locations were marked across the height of one of the blocks (that mimic the side wall of the actual device). The contact angle measurements were performed for blocks of different heights (4 to 8 mm). Blood droplet of volume <4 μl was placed at these marks in different trials and the contact angles of whole blood on PDMS surface were measured using goniometer (HOLMARC, Kochi, India).

In blood plasma separation studies, sample blood was introduced at the inlet of the device using a micropipette. The flow of blood in the vertical channel and the sedimentation of blood cells were observed and captured using a USB camera (Dino-Lite, AnMoElectronics, Taipei) and Dinocapture 2.0 software. The location of the blood-plasma interface, plasma and blood menisci were obtained by counting the pixels of the image taken and comparing with the known fixed total height of the channel. The same method was adopted for observing the sedimentation of blood in the cylindrical well. Using an inverted microscope (Carl Zeiss Axiovert A1,Germany) coupled with a high-speed camera (FASTCAM SA3 model, Photron USA, Inc.) interfaced with PC via PhotronFastcam Viewer 3 software, the location of the capillary meniscus of blood flow in the horizontal channel was tracked for the validation of the theoretical model.

## Results and Discussions

In this section, first, flow of blood sample in the bottom channel of the device is studied. Next, sedimentation of RBCs in a well with blood samples of different dilutions is investigated. Then, to understand the effect of wettability of the well surface on plasma separation, as a result of oxygen exposure, contact angle measurements are performed. Further, experiments are carried out to determine the optimum dimensions of device – diameter and height of the well– to achieve maximum rate of plasma separation. The final device is then characterized for its purification efficiency, stability over time and analyte-compatibility. Finally, the device is integrated with a glucose detection strip to demonstrate on-chip diagnosis of analyte.

### Blood flow in a rectangular PDMS capillary channel

**Figure 3:**
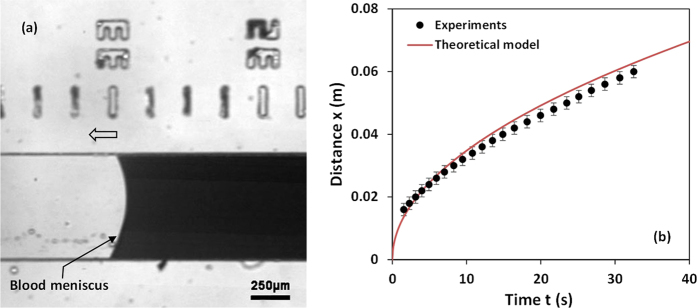
(**a**) Capillary meniscus of whole blood in bottom microchannel (**b**) Distance travelled by blood meniscus *x(t*) at different time instants *t*: comparison of theoretical model predictions (Supplementary Information) and experimental data (*n* = 6, error bars represent standard error of the mean).

A small drop of whole blood (5 μl) was introduced at the inlet of the device and the capillary flow in the bottom channel was observed (Fig. 3a). The location of the blood flow meniscus *x(t*) with time *t* obtained from experiments and predicted by the model (i.e. eqn. 11 in Supplementary Information) are presented in Fig. 3b. For the theoretical predictions, we take *m* = 0.035^33^ and *σ* = 0.055 N/m^34^. The value of *n* can vary between 0.7 and 1.0^35^, hence, an average value of *n* = 0.85 is taken. The variation of contact angle *θ* of the channel surface at different waiting time after oxygen plasma exposure was studied (Fig. S1 in Supplementary Information) and for a waiting time of 30 min, the contact angle θ was found to be 14°. Figure 3b shows that the predictions of the theoretical model match well with experimental data within 5%.

### Sedimentation of blood in a cylindrical well

**Figure 4:**
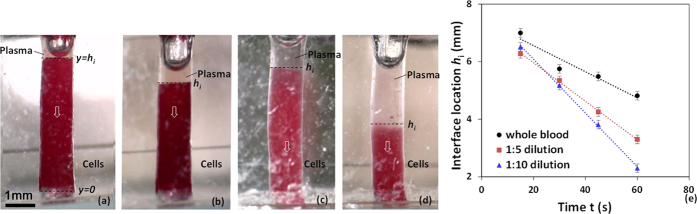
Sedimentation of RBCs in a cylindrical well, diameter 1.5 mm, height 6.0 mm (**a**) whole blood at 30 min and (**b**) whole blood at 60 min and (**c**) 1:10 diluted blood, 30 min (**d**) 1:10 diluted blood, 60 min (**e**) Location of interface *h*_i_ at different time instants for different dilution (*n* = 6, error bars represent standard error of the mean).

**Figure 5:**
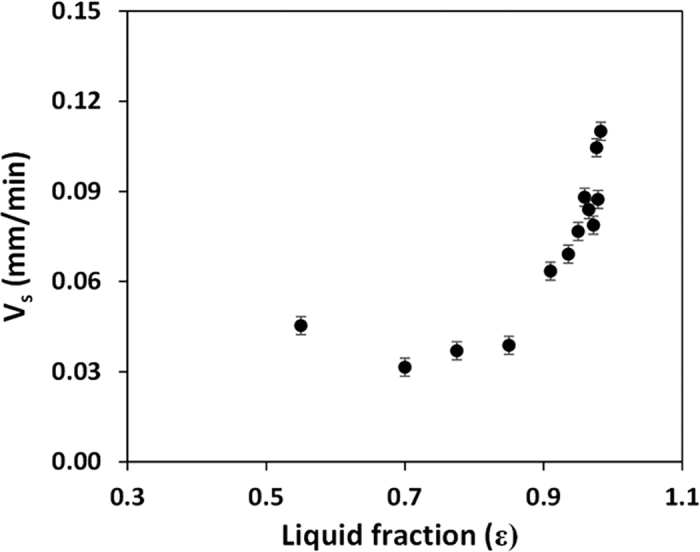
Variation of sedimentation velocity with liquid concentration ε (*n* = 6, error bars represent standard error of the mean).

Sedimentation of blood at different dilutions was studied by filling a cylindrical well of diameter 1.5 mm and height 6.0 mm with sample blood. Figure 4(a–d) show experimental images of the cell-plasma separation at two different time instants, 30 min and 60 min for whole blood and 1:10 diluted blood. The change in the total volume observed inside the well in the images may be due to the evaporation of plasma (since 95% water present in plasma) during the 30 min duration between the two time points. The location of the interface between the plasma and the sample blood with time is depicted in Fig. 4e, which shows that sedimentation process is faster (evident from higher slope) at higher dilutions. From the location of the interface at different time instants, the sedimentation velocity is obtained. The variation of sedimentation velocity with liquid concentration by volume ε at different dilutions is shown in Fig. 5. As observed, the sedimentation velocity increases with increase in ε, which is in agreement with eqn. 16 (Supplementary Information). It is interesting to note that higher sedimentation velocity was observed (from repeated experiments) in case of whole blood i.e. ε = 0.54 when compared to diluted blood with ε = 0.7, which cannot be explained by Steinour’s model^36^. The possible reason for this anomalous behaviour is that the cell aggregation which takes place in whole blood giving rise to a higher sedimentation velocity is absent when the blood sample is diluted with PBS^37^. As compared to the whole blood, in the low dilution range, with increase in ε, rouleaux starts to disperse and mass of these aggregations starts to reduce, which give rise to lower sedimentation rate. Derivation showing the correlation of sedimentation velocity (*V*_*s*_*f(ε*) in eqn. 16, Supplementary Information) in terms of the liquid concentration ε is given in Supplementary Information.

### Contact angle of the inner surface of the well

**Figure 6:**
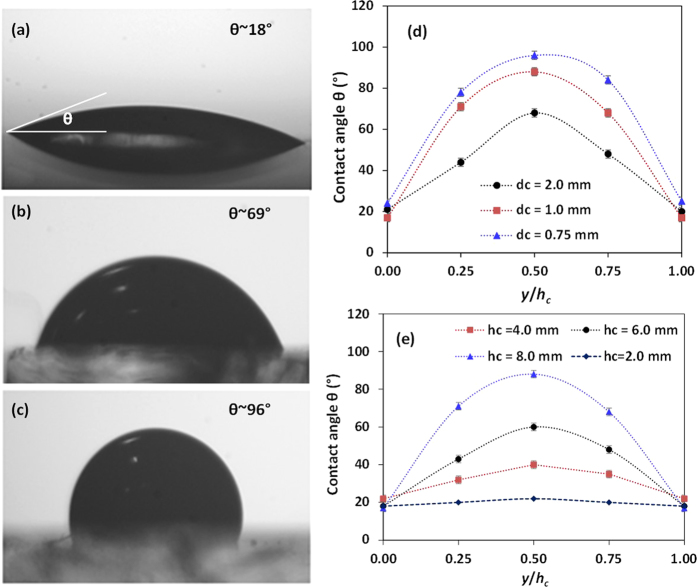
Images showing the contact angle of a blood droplet, 4 μl at (**a**) directly exposed channel edge, *y* = 0 (**b**) midpoint between centre and edge, *y* = 0.25 *h*_c_ (**c**) centre of the inner surface, *y* = 0.5*h*_c_, gap *d*_c_ = 1.5 mm, height *h*_c_ = 6 mm, Variation of contact angle with (**d**) different gaps between PDMS blocks, *h*_c_ = 8 mm (**e**) different height, gap *d*_c_ = 2.0 mm (*n* = 6, error bars in the plots represent standard error of the mean).

**Figure 7:**
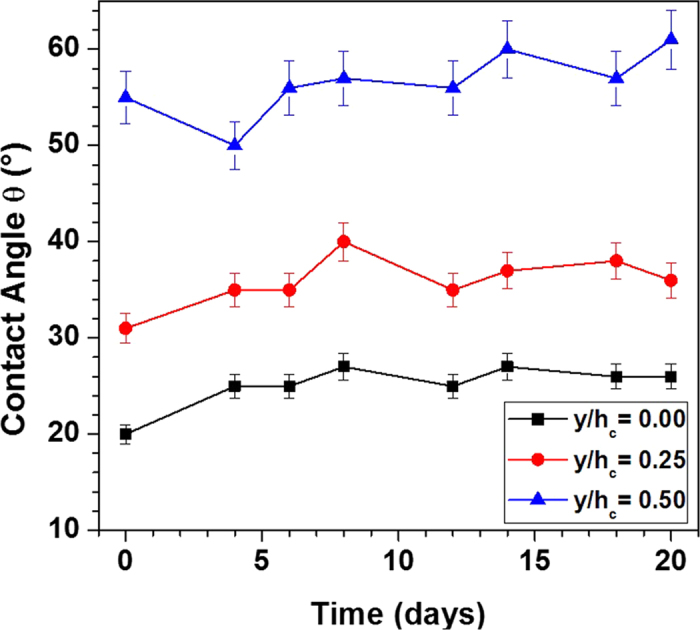
Variation of contact angle at three locations on the side wall (*y/hc* = 0, 0.25, 0.5) with time after storing in water under vacuum (*n* = 6, error bars represent standard error of the mean).

To determine the variation of contact angle on the inner surface of the vertical cylindrical channel (well), the experimental conditions are simulated as discussed in device fabrication and experimental setup section. Contact angle was measured at five different locations along the height of the side wall. The images showing the contact angle of sample blood drops at three different points (*y* = 0, 0.25*h*_c_ and 0.5*h*_c_) after 45 min of oxygen plasma exposure (11.0 W for 2 min) are shown in Fig. 6(a–c). It is clearly observed that the ends of the well which are well exposed to oxygen plasma have least contact angle of 18° whereas the middle portion of the well inner surface is least exposed and hence has the maximum contact angle of 96° indicating hydrophobicity. Since a smaller gap is available for the penetration of oxygen plasma into the well, the middle portion of the well receives only a limited amount of oxygen plasma^38^. Hence, even if the ends of the wells are hydrophilic, the middle portion can still retain its hydrophobicity. The variations of contact angle of the inner surface of the well along its height and the effect of the diameter and height of the well on the contact angle variation are presented in Fig. 6(d and e). The results show that the contact angle of the inner surface of the well at its middle portion reduces with increase in the well diameter and decrease in the well height due to higher penetration of the oxygen plasma and better exposure of the inner surface of the well. The contact angle of the inner surface of the well can be further controlled by adjusting the exposure time and chamber pressure (Fig. S2 in Supplementary Information). To retain hydrophilicity, PDMS blocks (as shown in Fig. 2b) with *h*_c_ = 4 mm and *d*_*c*_ = 1 mm were stored in DI water under vacuum^32^. Contact angles were measured at three different locations (*y/h*_*c*_ = 0, 0.25, 0.5) along the side wall on different days (0–20 days) after the exposure. From Fig. 7, we can observe that the hydrophilicity and the gradient can be maintained by using this method.

### Separation of plasma in the well of the device

**Figure 8:**
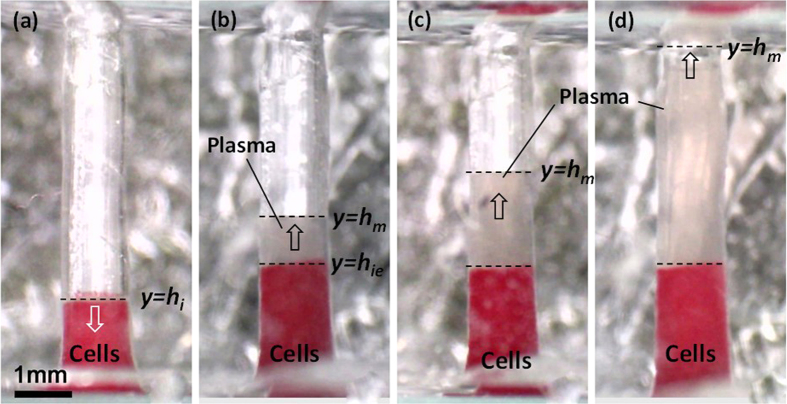
Images showing the separation and rise of plasma in the well, 1.5 mm diameter and 6.0 mm height at (**a**) 3 min, (**b**) 10 min (**c**) 20 min and (**d**) 30 min, plasma meniscus *h*_*m*_ and cell-plasma interface *h*_*i*_ are shown.

As illustrated in device description and principle, at a particular location along the height of the well, due to lower velocity (as a result of higher contact angle), plasma would no longer be able to carry the RBCs upward and thus starts to separate from the cells. The RBCs accumulate and form a self-built in filter and sediment while the plasma get separated and moves slowly upward. Figure 8(a–d) shows the optical images of the plasma separation at different time instants after the blood sample was introduced at the inlet of the device with a well of 1.5 mm diameter and 6.0 mm height. The results show that plasma separation starts at 3.0 min after the blood was first introduced at the device inlet. On the other hand, in the static sedimentation experiments, the plasma separation is initiated only after 10 min. This clearly shows the effect of wettability gradient in the well on the plasma separation. However, shear gradients under flowing conditions of the non-Newtonian blood may also play a role.

**Figure 9:**
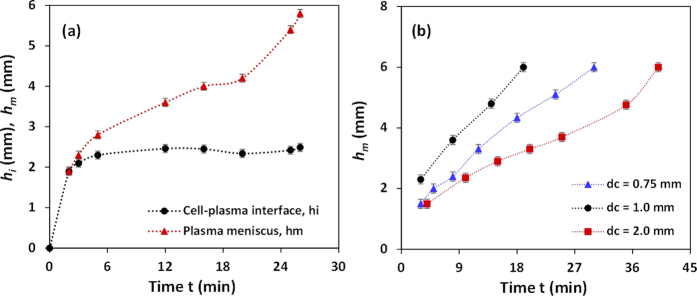
(**a**) Variation of the location of plasma meniscus *h*_*m*_ and cell-plasma interface *h*_*i*_ at different instants of time, *d*_*c*_ = 1.5 mm (**b**) variation of location of plasma meniscus h_m_ in wells of different diameters *d*_*c*_, *h*_*c*_ = 6.0 mm (*n* = 6, error bars represent standard error of the mean).

Figure 9(a) shows the locations of the cell-plasma interface *h*_*i*_ and plasma meniscus *h*_*m*_ at different instants of time during the plasma separation process using whole blood. We observe that at smaller time instants (*t* < 3 min) i.e. at the bottom end of the well, due to higher hydrophilicity of the wall, a higher velocity (higher slope in the *y(t*) versus *t* curve) of the cell-plasma interface is observed. Due to the increasing contact angle, the velocity is significantly reduced for *t* > 3 min and the plasma separation starts due to the formation of ‘self-built in filter’ and sedimentation. At the middle portion of the well, due to higher contact angle, the capillary force becomes inadequate to drive the cells that have much higher viscosity. The cells accumulate to form a ‘self-built in filter’ which helps in the filtration of the plasma and sedimentation of the cells. The location of the cell-plasma interface remains fixed (*h*_*i*_ = *h*_*ie*_, i.e. the equilibrium cell-plasma interface location) after *t* > 10 min and only the plasma meniscus moves upward due to much lower viscosity of plasma. Figure 9(a) also shows that the velocity of the plasma meniscus increases again when it approaches the top end of the well due to lower contact angle of the inner wall at this region. In this case, the plasma reaches the top end and rapidly moves into the top channel (due to much lower dead volume of the top channel as compared to the well) at *t* = 25 min and 6.0 μl of plasma is obtained.

For a fixed well height of 6.0 mm, the effect of the well diameter *d*_c_ on the plasma separation was studied. As discussed in the previous section, the contact angle is lower for a well of higher diameter due to better exposure of the inner surface of the well to oxygen plasma thus higher plasma velocity is expected. However, from theory of capillary flows it is known that the velocity of the capillary rise is inversely proportional to the well diameter. Due to the trade-off between these two competing effects, the velocity of the plasma meniscus was found to be maximum for a well diameter of 1.0 mm indicating faster rate of plasma separation, as shown in Fig. 9(b). For wells of diameter 1.5 mm (Fig. 9a) and 2.0 mm (Fig. 9b), the velocity is lower and therefore lower initial velocity and lower momentum in the less hydrophilic region in the well results in slower rise of plasma meniscus in this region. On the other hand wells of diameter 0.75 and 1.0 mm show comparatively faster rise of plasma meniscus in this region. The rise in plasma meniscus in well of 0.75 mm diameter is slower than 1.0 mm due to the larger contact angles in the former case as seen in Fig. 6d. It is interesting to note that irrespective of the well diameter in the range 0.75 to 2.0 mm, the plasma separation is initiated within 3 to 5 min of introduction of the sample blood at the inlet (minimum value of *h*_*m*_ for each case is equal to *h*_*ie*_) as shown in Table 1.

**Figure 10:**
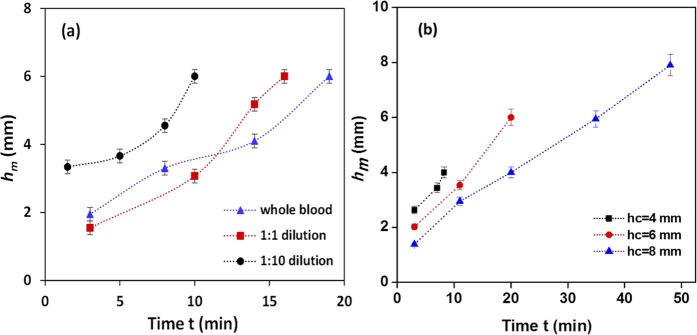
(**a**) Location of the plasma meniscus *h*_*m*_ at different instants of time *t* for different dilutions (**c**) Location of the plasma meniscus at different instants of time *t* for different channel heights *h*_*c*_ (*n* = 6, error bars represent standard error of the mean).

For a well of diameter 1.5 mm and height 6.0 mm, the effect of dilution (1:1, 1:5 and 1:10) on the cell plasma separation was studied. As shown in Fig. 10(a), at 1:1 dilution, the sedimentation happens faster thus the slope of the curve is higher as compared to whole blood. At this dilution, due to faster sedimentation (Fig. 5 and eqn. 16 in Supplementary Information), the plasma meniscus reaches the top of the well sooner as compared to that in case of whole blood. It is observed that the plasma separation starts early (at 2.0 min) for higher dilution ratios (1:10) as compared to whole blood (3.0 min), as shown in Fig. 10(a). At much higher dilutions (1:10), due to lower viscosity the diluted sample blood rises faster inside the well, thus even if the sedimentation is faster in a highly diluted blood sample, the separated plasma volume (i.e. difference between the plasma meniscus *h*_m_ and the cell plasma interface *h*_i_) is lower as compared to the whole blood case as given in Table 1.

For a well diameter 1.5 mm, the effect of well height on the plasma separation was studied. For a smaller well height, the inner surface of the well can get well exposed to oxygen plasma to provide lower contact angle, which leads to faster separation and rise in blood plasma as shown in Fig. 10(b). Thus, as shown in Table 1, in case of a 4 mm height well, the plasma meniscus reaches the top of the well much faster (within 10 min) as compared to that in case of wells of higher heights (48 min in case of 8 mm high well). The experiments with wells of different heights show the effect of wettability gradient on the volume of plasma obtained. Experiments with wells of height 2.0 mm where the wettability gradient is almost negligible (Fig. 6e) did not show any blood plasma separation (Table 1, last row). Figure 6(e) shows the differences in wettability gradient in wells of different heights. As observed, when the height is smaller (e.g. 4.0 mm), the wettability gradient (i.e. *dθ/dy*) is smaller as compared that for a well of larger height (e.g. 8.0 mm). Table 1 (last row) shows that volume of plasma increases when the well height increases implying the role of contact angle gradient in plasma separation. Due to more capacity (channel volume), the total volume of plasma collected is higher in the case of 2.0 mm diameter well but this is not of importance in the proposed device as long as the top channel is completely covered with plasma. However, due to higher plasma separation rate, the plasma meniscus reaches the top of the well faster in case of 1.0 mm diameter well and moves into the top channel quickly thus could reduce the overall detection time.

Therefore, from these observations, we select a well of diameter 1.0 mm and height 4.0 mm for our device, so as to obtain plasma in the top channel faster to minimize detection time.

**Figure 11:**
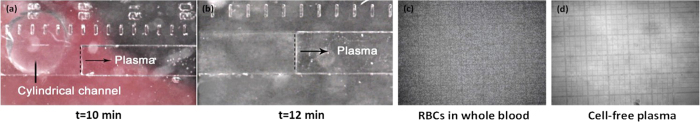
(**a**–**b**) Optical images showing the flow of separated plasma in the top channel at two different locations *x* = 1.0 mm and 10 mm at time *t* = 10 min and 12 min, respectively (**c–d**) whole blood and plasma on the haemocytometer grid.

For the range of well diameters and heights studied here, since the separation takes place inside the well, we always obtain pure plasma in the top channel. The flow of the separated plasma in the top channel was captured using the microscope at two different locations *x* = 1.0 mm and 10 mm at time *t* = 10 min and 12 min, respectively, are shown in Fig. 11(a) and (b). At *t* = 15 min, 2.0 μl of plasma was obtained from the whole blood sample.

The separated plasma was collected at the device outlet and the purification efficiency was measured using an Improved Neubaeur Haemocytometer (Marienfeld-Superior, Germany), a gridded device generally used to count cells. An aliquote of 10 μl of the sample was added to each of its chamber and viewed under microscope. The number of cells in 5 squares of side 1.0 mm was counted on both the chambers and averaged. The % *Purification Efficiency* was calculated from the experiments as follows,


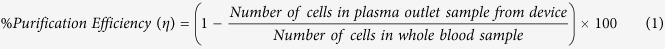


**Figure 12:**
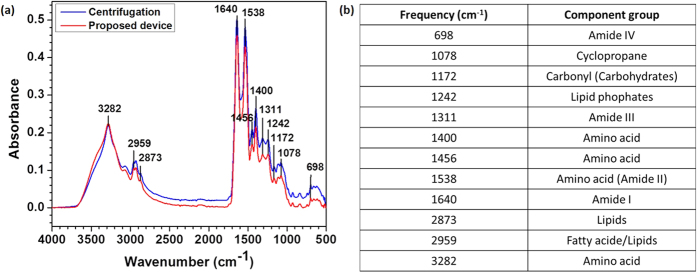
(**a**) ATR-FTIR analysis and comparison of plasma samples from proposed device and centrifugation (**b**) Table shows the component groups corresponding to the characteristic bands.

The optical images of the whole blood and plasma on the haemocytometer grid are shown in Fig. 11(c) and (d), respectively, which evidently shows that the number of cells at the plasma outlet of the device is significantly lower. The purification efficiency was found to be 99.9%. Further, to determine the residual composition of the plasma after the separation, ATR-FTIR analysis was performed and compared with that for the plasma obtained from centrifugation as shown in Fig. 12. The tabulated data shows the list of characteristic bands in the infrared spectrum corresponding to different biomolecules, eg., carbohydrates, proteins, lipids and fatty acids. The similar peaks found in the plasma samples from both centrifugation and the proposed device indicate that biomolecule do not get filtered along with RBCs during the separation process but are present in the plasma. These characterization experiments on the plasma indicate that the plasma obtained using the device is comparable to that obtained using conventional centrifuge and can be used for detection of analytes present in the plasma. Further, the device was filled with DI water, kept under vacuum and checked for plasma separation after 15 days. On comparison with the freshly prepared device, it was observed that the performance of the device in terms of plasma volume, separation time and purification efficiency remains unaffected.

### Detection of glucose

**Figure 13:**
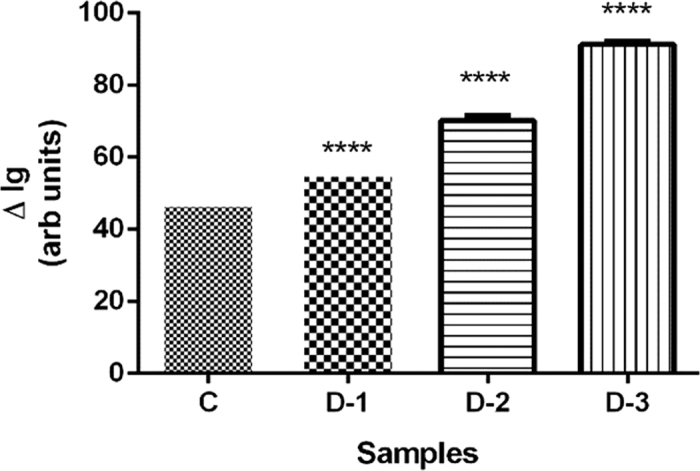
Comparison of difference in the gray scale intensities Δ*I*_*g*_ of glucose detection strip on reaction with plasma of non-diabetic and diabetic patients (*n* = 3, error bars represent standard error of the mean. For statistical significance, one-way analysis of variance (ANOVA) was performed on these values followed by Dunnett’s Multiple Comparison Test for multiple comparison of D-1, D-2, D-3 with control sample (C) with statistical significance threshold set at 0.05 (*p* < 0.05)).

Glucose was detected in the plasma separated using the proposed device by employing functionalized surfaces from commercial glucose strips (Accu-Chek Extra Care, Roche Diagnostics India Pvt. Ltd). Whole blood samples from normal and diabetic patients were introduced into different devices. The plasma get separated in the cylindrical well and flows in the top channel. The glucose detection strips were integrated with the top channel during bonding. On reaction of the glucose in the separated plasma with the enzyme functionalized surface on the strip, depending on the concentration of glucose in the plasma, the colour of the strip changes from yellow to green/blue, which corresponds to specific RGB intensity values. The captured images of these strips for each sample were converted to gray scale and the gray scale intensities *I*_*g*_ were measured for each sample using ImageJ software. The difference between *I*_*g*_ of glucose-reacted and unreacted surface on the strip, Δ*I*_*g*_ was used for comparison of the plasma samples of diabetic donors (D-1, D-2, D-3) and non-diabetic donor (C). The actual glucose concentrations in individual blood samples were obtained with the help of the standard procedure (GOD-POD Enzymatic assay) in IIT Madras Institute Hospital for validation and the values of C, D-1, D-2 and D-3 were found to be 84, 101, 139 and 183 mg/dl, respectively. The results agree well qualitatively with the Δ*I*_*g*_ measured for the strips treated with the plasma obtained using our device. A correlation between glucose concentration and Δ*I*_*g*_ is observed which shows that Δ*I*_*g*_ is directly proportional to the glucose concentration in blood, as depicted in Fig. 13. The experiments demonstrated in the paper is a proof-of-concept for on-chip detection of glucose using the proposed device and to provide a quantitative evidence for the correlation between actual glucose concentration and the gray scale intensities Δ*I*_*g*_ developed in the strip. In practical applications, the glucose concentration values can be obtained by matching the colour change in the strip with the colour code or reading the RGB/Ig with a hand-held instrument as usually done with Accu-Chek glucose strips.

## Conclusions

We reported a self-powered, simple and low cost microfluidic device driven by capillary force for the separation of plasma from whole blood by exploiting wettability gradient and sedimentation effects. Contact angle measurements of the inner surface of the well (cylindrical channel) showed a lower contact angle (~20°) at the top and bottom ends and much higher contact angle (>90°) at the central region. The effects of the well diameter and height on the contact angle variation were studied which showed that the contact angle gradient is higher in case of chamber of smaller diameter and larger height. The plasma separation process in the well with a wettability gradient was experimentally investigated by measuring the variation of the location of the plasma meniscus and the cell-plasma interface at different time instants. The effect of the well diameter (0.75 mm, 1.0 mm and 2.0 mm) and height (4.0 mm, 6.0 mm and 8.0 mm) on the rate of plasma separation was studied which showed higher plasma separation rate for a well of 1.0 mm diameter and 4.0 mm height. By using a well of 1.0 mm diameter and 4.0 mm height, 2.0 μl of plasma was obtained from whole blood (<10 μl) in 15 min. The system can be parallelized to obtain higher volume of plasma, if required. The purification efficiency was found to be 99.9%, which is comparable with that obtained using conventional centrifugation. The wetting property of the channels can be maintained by storing in DI water under vacuum over three weeks. The performance of the device was found to be unaffected in terms of plasma volume, separation time and purification efficiency during this period. The higher plasma separation rate and purification efficiency shows the potential of the proposed device for point-of-care diagnostics.

## Materials and Methods

### Blood sample

Whole blood was collected from healthy donors and diabetic donors at Institute Hospital (IIT Madras) in vacutainers with 7.2 mg K2 Ethylene diaminetetraacetic acid (EDTA) (BD, New Jersey, USA) after obtaining ethical clearance. Phosphate Buffered Saline (PBS) (Sigma Aldrich, Bangalore, India) was used for dilution of the blood, when required.

All experiments were performed in accordance with the relevant guidelines and regulations of the Institute Ethics Committee of Indian Institute of Technology Madras. The experiments were approved by the Institute Ethics Committee of Indian Institute of Technology Madras and written informed consent was obtained from all subjects.

### Quantification of blood plasma separation

The performance of the proposed device for blood plasma separation was quantified in terms of purification efficiency. To measure purification efficiency, the concentrations of cells in whole blood and the plasma obtained from the device were measured with the help of Improved Naeubaer Haemocytometer (Marienfeld, Germany) and used for determining purification efficiency of the device as given in eqn (1).

#### Analysis by ATR-FTIR

A drop of plasma (1 μl) sample from centrifugation and proposed device was placed on the crystal of the ATR-FTIR equipment (Alpha FTIR Spectrometer, Bruker Corporation, United States) and the infrared spectrum was recorded in the frequency range 4000–500 cm^−1^.

#### Statistical analysis

Experiments were repeated for *n* = 6 trials with fresh blood sample and PDMS device for every trial. Error bars in the plots represented standard error of the mean (SEM). All statistical analyses were performed using OriginPro 8 (OriginLab Corporation, USA). For detection of glucose, Δ*I*_*g*_ measurements were repeated for *n* = 3 trials at different locations on the same strip. One-way analysis of variance (ANOVA) was performed on these values followed by the multiple comparison of the diabetic samples (D-1, D-2, D-3) with the control sample (C) by Dunnett’s Multiple Comparison Test with statistical significance threshold set at 0.05 (p < 0.05) using GraphPad Prism software (GraphPad Software, Inc., La Jolla, CA).

## Additional Information

**How to cite this article:** Maria, M. S. *et al*. Capillary flow-driven microfluidic device with wettability gradient and sedimentation effects for blood plasma separation. *Sci. Rep.* **7**, 43457; doi: 10.1038/srep43457 (2017).

**Publisher's note:** Springer Nature remains neutral with regard to jurisdictional claims in published maps and institutional affiliations.

## Supplementary Material

Supplementary Information

Supplementary Information

## Figures and Tables

**Table 1:** Table showing *h*_ie_, initiation of plasma separation and the time for the plasma meniscus to reach the top channel for the different parameters tested.

Fixed parameters	Variable parameters	h_ie_ (mm)	Initiation of plasma separation T_0_ (min)	Time for plasma meniscus to reach top channel T_f_ (min)	Volume of plasma (μl)
*h*_*c*_ = 6mm, Whole blood	*d*_*c*_ = 0.75 mm	1.5	3.0	30.0	2.0
	*d*_*c*_ = 1 mm	2.3	3.0	19.0	3.0
	*d*_*c*_ = 2 mm	1.5	5.0	40.0	12.0
*h*_*c*_ = 6mm, *d*_*c*_ = 1.5 mm	Dilution – Whole blood	1.95	3.0	19.0	7.1
	Dilution – 1:1	1.55	3.0	16.0	7.8
	Dilution – 1:10	3.34	2.0	10.0	4.7
*d*_*c*_ = 1.5 mm, Whole blood	*h*_*c*_ = 2 mm	—	—	—	—
	*h*_*c*_ = 4 mm	2.6	3.0	8.2	2.4
	*h*_*c*_ = 6 mm	2.0	3.0	20.0	7.0
	*h*_*c*_ = 8 mm	3.0	3.0	48.0	8.8
