# Supplementary material for: Capillary flow-driven microfluidic device with wettability gradient and sedimentation effects for blood plasma separation
Source: Sci Rep. 2017 Mar 3;7:43457. doi: 10.1038/srep43457 (PMC5335260; doi:10.1038/srep43457)
Supplement: Supplementary Information [file srep43457-s1.docx]

**SUPPLEMENTARY MATERIAL**

**Capillary flow-driven microfluidic device with wettability gradient and sedimentation effects for blood plasma separation**

M. Sneha Maria^1,2^, P. E. Rakesh^1^, T. S. Chandra^2^, A. K. Sen^1,*^

^1^Department of Mechanical Engineering, Indian Institute of Technology Madras, Chennai-600036, India

^2^Department of Biotechnology, Indian Institute of Technology Madras, Chennai-600036, India

*Both first and second authors have equally contributed to the work*

^*^Author to whom correspondence should be addressed. Email: [ashis@iitm.ac.in](mailto:ashis@iitm.ac.in)

**Fabrication of master mold and PDMS device**

The mask design of the device was prepared using AutoCAD LT 2008, and was printed on a flexi mask at 40000 dpi (Fineline Imaging, USA). First, silicon wafer (Semiconductor Technology and Application, Milpitas, USA) was cleaned with a mixture of hydrofluoric acid solution and deionised water at 1:10 ratio and then baked in oven for 2 min at 120 ˚C. SU8 2075 (MicroChem Corp, Newton, USA), the negative photoresist, was spun coated onto the Si wafer at 1100 rpm for 30 s at acceleration 200 rpm/s. It was then soft baked at 65 ˚C for 7 min and hard baked at 95 ˚C for 30 min. Further, it was exposed to UV light (J500-IR/VISIBLE, OAI mask aligner, CA, USA) through the flexi mask and then baked at 65 ˚C for 5 min and 95 ˚C at 10 min. Finally, the exposed pattern was developed in SU8 developer solution to obtain the SU8 master. The SU8 master was then placed inside oven at 120 ˚C for 30 min. Besides the straight channels, scales of least count 0.1 mm, marked from 0 to 60 mm are also printed in the master to observe the meniscus location with time. A photograph of the proposed blood plasma separation device is depicted in Fig. 2a. To fabricate the PDMS device, PDMS monomer and curing agent (Sylgard 184, Silicone Elastomer kit, Dow Corning, USA) were mixed at a ratio 10:1 by weight, degassed and poured onto the SU8 master. It was then cured by placing in a vacuum oven at 65 ˚C for 3 h. Further, it was peeled off from the SU8 master and cut to shape.

**Theory**

**Capillary flow of blood: power–law model**

Cito *et al*, 2012 [1] have developed a simple relation for power law non-Newtonian fluids by generalizing Lucas-Washburn model. Here, we derive a relationship for the capillary flow of blood from the Ostwald–de Waele power law model. The model can be used to predict the shear–thickening or shear–thinning behaviour of non–Newtonian liquids. In this case, the effective dynamic viscosity is given by

(1)

where is the magnitude of the strain rate tensor and *m* and *n* are constants depending on the properties of the liquid. Since the aspect ratio of the microchannel is higher, assuming the variation of velocity along $y$–direction to be small, i.e.,, eqn.1 can be written as

$\mu=m\left|\frac{\partial u}{\partial z}\right|^{n-1}$ (2)

where *u* is the velocity. Further, assuming the flow to be fully developed and using the Poiseuille equation, we get

$\frac{\partial}{\partial z}\left(m\left|\frac{\partial u}{\partial z}\right|^{n-1}\frac{\partial u}{\partial z}\right)=\frac{\partial p}{\partial x}$ (3)

where *p* is the pressure. By integrating eqn.3 with no–slip boundary conditions at the top and bottom walls, i.e.,$u(z=0)=0=u(z=h)$, the expression for velocity is found as

$u(z)=\frac{n}{2(n+1)}\left[\frac{1}{2m}\left(-\frac{\partial p}{\partial x}\right)\right]^{\frac{1}{n}}\left(h^{1+\frac{1}{n}}-|2z-h|^{1+\frac{1}{n}}\right)$ (4)

where *h* is the channel height. Thus, the flow rate $q$ can be obtained as

$q=w\int_{0}^{h} u(z) \text{d}z$ (5)

where$w$is the width of the channel. From eqn. 4 and 5, the flow rate $q$ is found as

$q=\frac{n}{2(1+2n)}\left[\frac{1}{2m}\left(-\frac{\partial p}{\partial x}\right)\right]^{\frac{1}{n}}wh^{2+\frac{1}{n}}$ (6)

The flow rate $q$ can also be expressed as

$q=wh \frac{\text{d}x}{\text{d}t}$ (7)

Now, from eqn. 6 and 7, we can write

$wh \frac{\text{d}x}{\text{d}t}=\frac{n}{2(1+2n)}\left[\frac{1}{2m}\left(-\frac{\partial p}{\partial x}\right)\right]^{\frac{1}{n}}wh^{2+\frac{1}{n}}$ (8)

The driving pressure is the Young- Laplace pressure drop$\left(\Delta p\right)$across the meniscus. By assuming the pressure at the ends of the microchannel to be atmospheric [2], we can write

$-\frac{\partial p}{\partial x}=\frac{\Delta p}{x}$ (9)

The Young-Laplace pressure drop$\Delta p=\frac{2\sigma\cos\theta}{h}$ ,$\sigma$is the surface tension of the liquid and $\theta$ the contact angle. Therefore, eqn. 8 is written as,

$\frac{\text{d}x}{\text{d}t}=\frac{n}{2(1+2n)}\left[\frac{1}{2m}\left(\frac{2\sigma\cos\theta}{x}\right)\right]^{\frac{1}{n}}h$ (10)

If we rearrange the terms in the above equation and integrate the left hand side from$0$ to $x$ and the right hand side from $t_{0}$to$t$, we get

$x(t)=\left[\frac{n+1}{2(2n+1)}\left(\frac{\sigma\cos\theta}{m}\right)^{\frac{1}{n}}h\right]^{\frac{n}{n+1}}(t-t_{0})^{\frac{n}{n+1}}$ (11)

The above eqn is of the form$x(t)=A(t-t_{0})^{\gamma}$ where $\gamma=\frac{n}{n+1}$and the constant$A$, analogous to the Washburn’s constant [3] for Newtonian liquids, is given by:

$A=\left[\frac{n+1}{2(2n+1)}\left(\frac{\sigma\cos\theta}{m}\right)^{\frac{1}{n}}h\right]^{\frac{n}{n+1}}$ (12)

The eqn. 11 is used later to predict the location of the meniscus at various instants of time and validated by comparing the model predictions with the experimental data.

**Sedimentation of RBCs**

The viscous drag force experienced by a spherical particle falling down in a medium is given by$F_{D}=6\pi\mu rV_{s}$where $r$ is the radius of the particle and$\mu$ is the viscosity of the medium. The terminal velocity attained by the particle due to the opposing buoyancy and gravitational forces is given as

$V_{s}=\frac{2g\left(\rho_{s}-\rho_{f}\right) r^{2}}{9\mu}$ (13)

where $V_{s}$is the Stoke’s velocity and$\rho_{s}$and$\rho_{f}$ are the densities of the solid and fluid components of the suspension, respectively. The erythrocyte sedimentation rate can also be obtained directly from the Stokes equation as in [4]. However, it does not account for the hematocrit concentration, viscosity of suspending fluid at different dilutions, cell aggregation and rouleaux formation with increase in hematocrit. The theoretical model has been compared with the experiment with blood suspended in a 1ml syringe. These parameters could have led to the error on comparison with the theoretical model. From literature, it is known that for a suspension of particles, the particles settling down will replace equal volume of liquid which exerts an upward force on the particles [5] which slows down settling of particles. Thus the settling velocity is inversely proportional to concentration of particles.

Assuming uniform distribution of particles in the suspension such that the flow space is uniform, Steinour [6] modelled the sedimentation velocity of uniform spherical particles as a function of concentration and flow space as

$V=V_{s}\varepsilon^{_{2}}\varphi\left(\varepsilon\right)$ (14)

where$\varepsilon$ is the liquid concentration i.e. (1- concentration of RBCs) and $\varphi\left(\varepsilon\right)$ is the function which defines the effect of flow space, which can vary from 0 to 1. Similarly, blood can be considered as a uniformly distributed suspension of RBCs in plasma if we ignore the aggregate formation. By neglecting other effects, blood can be modelled in the same format with function of flow space being different as compared to that in case of spherical particles. However, the effect of dilution using a buffer (for example PBS) on the sedimentation velocity due to the change in the viscosity of the medium is not considered in eqn. 14. In order to account for the effect of the buffer we write

$V=V_{s}\varepsilon^{2}\varphi\left(\varepsilon\right) f_{\mu}\left(\varepsilon\right)$ (15)

where$f_{\mu}$is also a function of liquid concentration$\varepsilon$. In the limiting case, at infinite dilutions with a suitable buffer, the viscosity of the diluted sample will be closer to that of the buffer. Eqn.15 can be further simplified as

$V=V_{s}\varepsilon^{2}f\left(\varepsilon\right)$ (16)

Here, RBCs are assumed to be spherical particles of same volume as that of the actual RBCs.

We use experimental data for developing a correlation for the unknown term$V_{s}f\left(\varepsilon\right)$ in eqn. 16 in terms of the liquid concentration ε. The variation of$V_{s}f\left(\varepsilon\right)$with ε is presented in Fig. S1 (inset). By using curve fitting, a correlation is found as follows,

$V_{s}f\left(\varepsilon\right)=0.1063 \varepsilon^{4.08}$ (17)

In a limiting case, at infinite dilutions, $f\left(\varepsilon\right)=1$ [6], so $V_{s}$=0.1063 mm/min, which compares well with the Stoke’s velocity$V_{s}$=0.1112 of RBC in PBS predicted using eqn. 13, taking the values of $\rho_{s}$= 1100 kg/m^3^ and$\rho_{f}$=995 kg/m^3^ [7], respectively and viscosity of PBS is taken as 1.0 cP [8], RBC is assumed as a sphere of same volume as the actual RBC i.e.167 μm^3^ [9] and the diameter is found to be approximately 5.5μm. Using the above correlation for $V_{s}f\left(\varepsilon\right)$, eqn. 16 can be used to predict the sedimentation velocity of RBCs in a blood sample of known liquid concentration ε.

Fig. S1 Variation of sedimentation velocity with liquid concentration ε, inset shows variation of $V_{s}f\left(\varepsilon\right)$with ε (*n*=6, error bars represent standard error of the mean)


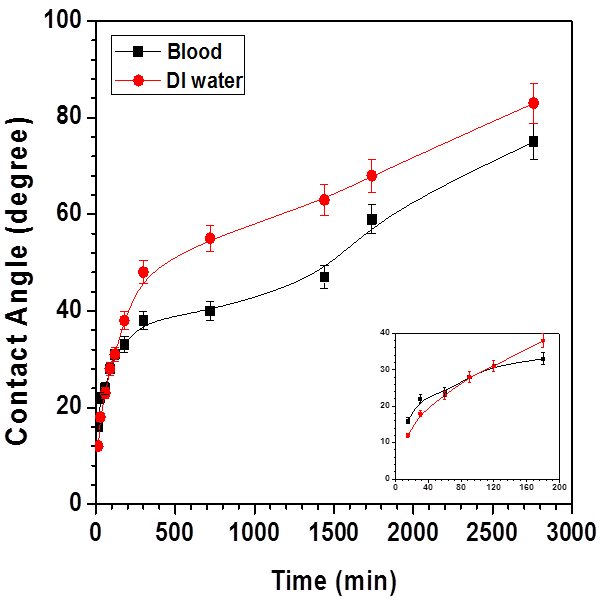


**Fig. S2** Variation of contact angle of water and blood with waiting time after oxygen-plasma exposure

**Fig. S3** Variation of contact angle of PDMS with the oxygen plasma exposure time and chamber pressure

Video: Shows the blood plasma separation process

## References

1. Cito, S., Ahn, Y.C., Pallares, J., Duarte, R.M., Chen, Z., Madou, M., Katakis, I., Visualization and measurement of capillary-­‐driven blood flow using spectral domain optical coherence tomography. *Microfluidics and Nanofluidics*, 13, 227–237 (2012). doi:10.1007/s10404‐012‐0950‐6.
2. Anoop R. and Sen A. K., Capillary flow enhancement in rectangular polymer microchannels with a deformable wall. Phys. Rev. E, 92, 013024 (2015).
3. Washburn E., The dynamics of capillary flow. Phys. Rev., 273:17 (1921).
4. Richardson J. F. and Zaki W. N., Sedimentation and Fluidization: Part 1. Icheme, 75, S82-S100 (1975).
5. Kuroda C. et al., Microfluidic Sedimentation System for Separation of Plasma from Whole Blood. IEEE Sensors, 1854-1857 (2014).
6. Steinour H. H., Rate Of Sedimentation- Non Flocculated Suspensions Of Uniform Spheres. Industrial and Engineering Chemistry, 36, 618 (1944).
7. Heravi F. M. et al., Impact of biofluid viscosity on size and sedimentation efficiency of the isolated microvesicles, *Membrane Physiology and Biophysics*, **3**, 162, 1-6 (2012).
8. S. H. Tan, N. Nguyen, Y. C. Chua, T. G. Kang. Oxygen plasma treatment for reducing hydrophobicity of a sealed polydimethylsiloxane microchannel. Biomicrofluidics 4, 032204 (2010).
9. Chandran K. B., Biofluid Mechanics-The human Circulation, CRC Press.
